# Reduction of NADPH oxidase 4 in adipocytes contributes to the anti-obesity effect of dihydroartemisinin

**DOI:** 10.1016/j.heliyon.2023.e14028

**Published:** 2023-02-25

**Authors:** Hu Hua, Mengqiu Wu, Tong Wu, Yong Ji, Lv Jin, Yang Du, Yue Zhang, Songming Huang, Aihua Zhang, Guixia Ding, Qianqi Liu, Zhanjun Jia

**Affiliations:** a Department of Nephrology, Children's Hospital of Nanjing Medical University, Nanjing, China; b Nanjing Key Laboratory of Pediatrics, Children's Hospital of Nanjing Medical University, Nanjing, China; c Jiangsu Key Laboratory of Pediatrics, Nanjing Medical University, Nanjing, China; d Department of Child Health Care, Children's Hospital of Nanjing Medical University, Nanjing, China; e Department of Endocrinology, Children's Hospital of Nanjing Medical University, Nanjing, China

**Keywords:** Dihydroartemisinin, Obesity, NOX4, Adipocyte differentiation, Lipid accumulation

## Abstract

Artemisinin derivatives have been found to have anti-obesity effects recently, but the mechanism is still controversial. Herein, long-term DHA treatment in obese mice significantly reduced the body weight and improved glucose metabolism. However, short-term DHA treatment did not affect glucose metabolism in obese mice, suggesting that the improved glucose metabolism in mice with DHA treatment could be secondary to body weight reduction. Consistent with previous reports, we observed that DHA inhibited the differentiation of adipocytes. Mechanistically, DHA significantly reduced the expression of NADPH oxidase 4 (NOX4) in white adipose tissue (WAT) of mice and differentiated adipocytes, and using NOX4 siRNA or the NOX4 inhibitor GKT137831 significantly attenuated adipocyte differentiation. Over-expression of NOX4 partially reversed the inhibition effect of DHA on adipogenic differentiation of preadipocytes. In addition, targeted proteomics analysis showed that DHA improved the abnormality of metabolic pathways. In conclusion, DHA significantly reduced fat mass and improved glucose metabolism in obese mice, possibly by inhibiting NOX4 expression to suppress adipocyte differentiation and lipid accumulation in adipocytes.

## Highlights

• DHA improved glucose metabolism in obese mice possibly by ameliorating obesity.
• DHA attenuated lipid accumulation in white adipose tissues and differentiated adipocytes.
• DHA inhibited adipocyte differentiation possibly by inhibiting NOX4 expression.
• DHA improved the abnormality of metabolic pathways.

## 1 Introduction

Obesity is a rising global epidemic with a high risk for metabolic syndromes that include cardiovascular disease, type 2 diabetes mellitus, dyslipidemia, and even malignant tumors [[1], [2], [3]]. The current therapeutic approaches to obesity management principally include lifestyle modifications such as reducing food intake and increasing exercise, inhibiting intestinal lipid absorption, and undergoing weight-loss surgery [[4], [5], [6], [7]]. However, obese patients often face a rapid rebound in body weight after maintaining short-term weight loss using the above methods [8,9]. Therefore, using compounds derived from traditional Chinese medicine constitutes another option that can be applied to treat obesity and metabolic diseases due to their long-term effectiveness and safety [10].

During the development of obesity, excess calories accumulate as fats in adipocytes and lead to the growth and expansion of WAT [11], and adipocytes are generated through adipogenesis from specific precursor cells. Although the total number of adipocytes in lean and obese adults does not differ, approximately 10% of fat cells are renewed annually in adults [12]. Therefore, adipocyte differentiation is a vital process for both adipocytes and the development of obesity [13]. The process of adipogenesis requires an orchestrated multistep process controlled by the activation of key transcription factors that include peroxisome proliferator-activated receptor γ (Pparγ) and CCAAT/enhancer binding protein α and β (C/Ebpα, C/Ebpβ) [14], and the expression of fatty acid synthase (Fasn) is activated in the late phase of differentiation to promote adipogenesis [15].

Reactive oxygen species (ROS) are pervasive signaling molecules in biologic systems, and ROS generation and scavenging are tightly regulated to maintain homeostasis [16]. ROS in adipose tissue increases during the development of obesity, and there are accumulating evidences that implicate a tight regulation of adipogenesis by ROS [[17], [18], [19]]. Among the various enzymes responsible for ROS generation are mitochondrial electron transport chain complexes I and III, nitric oxide synthases (NOSs), CYP450 reductase, xanthine oxidase, and NADPH oxidases (NOXs); with NOXs comprising the only enzymes whose primary function is to generate superoxide/ROS [20]. Of the NOX family members, NOX4 is primarily expressed in adipocytes, is the major source of ROS production during adipocyte differentiation [21]. Moreover, the expression level of NOX4 represents a switch between proliferation and differentiation in preadipocytes [22].

Artemisinin is a nature product initially extracted from plant *Artemisia annua* and applied as an antimalarial drug. Interestingly, recent studies have shown that artemisinin and its derivatives exert potential anti-obesity effects [23,24] with the underlying mechanism(s) of action needing further exploration. Dihydroartemisinin (DHA) is an active metabolite of artemisinin widely used to treat malaria, and our *in vivo* experiments suggested that long-term DHA treatment improved high-fat diet (HFD)-induced obesity and that the improved glucose metabolism in the obese mice were secondary to body weight reduction. Further research showed that DHA inhibited the differentiation of adipocytes both *in vivo* and *in vitro*. In terms of mechanism, we demonstrated that DHA significantly reduced the expression of NOX4 in WAT and differentiated adipocytes, and the application of NOX4 siRNA or the NOX4 inhibitor GKT137831 significantly inhibited adipocyte differentiation. We additionally implemented ultra performance liquid chromatography tandem mass spectrometry (UPLC-MS/MS)-based DHA targetom analysis using DHA-treated preadipocytes during differentiation. Our results showed that a total of 85 proteins involved in metabolic pathways were conformationally changed after DHA treatment, providing additional evidence that DHA affected adipocyte metabolism.

## 2 Material and methods

### 2.1 Animal experiments

Six-week-old male C57BL/6J mice were purchased from Nanjing Medical University, maintained under a 12-h light/12-h dark cycle at 22 ± 2 °C and a relative humidity of 55 ± 10%, and provided food and water ad libitum. For the diet-induced obese (DIO) mouse model, mice were fed with a high-fat diet (HFD) (D12492, Research Diets, USA), and control mice were fed with a normal diet (ND) (Beijing Keao Xieli Feed Co., Ltd., China). For the long-term DHA-treatment experiment, mice were randomly distributed into the following four groups: a group fed with the ND, a group fed with the ND supplemented with DHA, a group fed with the HFD, and a group fed with the HFD supplemented with DHA. For the short-term DHA treatment experiment, mice were fed with HFD or HFD supplemented with the same dose of DHA as for the long-term treatment. For these experiments, we mixed DHA (D7439, Sigma) with an ND or HFD at a dose of 25 mg/kg/d. The body weights (BWs) of the mice were documented, and food intake of each mouse was recorded once per day for three consecutive days. At the end of the experimental period, the animals were sacrificed and adipose tissue and blood were collected for subsequent analyses. All animal experiments were performed according to the protocols approved by the Institutional Animal Care and Use Committee of Nanjing Medical University (IACUC14030112-1).

### 2.2 Glucose tolerance test (GTT) and insulin tolerance test (ITT) analyses

GTT and ITT experiments were conducted to evaluate the glucose metabolic rate of the correspondingly treated mice. For the GTT, mice were fasted overnight (from 5 p.m. to 9 a.m.), and fasting blood glucose was assessed (0 min). Then, 2 g/kg glucose was injected intraperitoneally (i.p.), and tail blood glucose was measured using a handheld glucometer (Ascensia Breeze, Bayer Company, Germany) at 15, 30, 60, 90, 120, and 150 min after glucose injection. For the ITT, mice were fasted for 4 h with free access to drinking water and the basal blood glucose levels were recorded (0 min), after which the mice received an i.p. injection of 0.75 units/kg insulin (NovoRapid, Novo Nordisk); and the glucose concentrations were determined at 15, 30, 60, and 90 min after insulin injection.

### 2.3 Quantitative real-time PCR (qRT-PCR)

The total RNA of tissues or cultured cells was extracted with TRIzol Reagent (Takara, Takara Biotechnology, Dalian, China). A total of 1 μg of RNA from each sample was subsequently reverse-transcribed to cDNA in a 20-μL reaction system using a PrimeScript RT reagent kit (Takara, Tokyo, Japan) in accordance with the manufacturer's protocol. The cDNA was then diluted three times and 2 μL of cDNA was used as a template for PCR adopting a two-step method. qRT-PCR amplification was conducted with a SYBR Green Master Mix (Vazyme, Nanjing, China) on an ABI 7500 Real-time PCR Detection System (Foster City, CA, USA) or HONGSHI Real-time PCR Detection System (Shanghai, China). The cycling conditions were 95 °C for 10 min, followed by 40 cycles of 95 °C for 15 s and 60 °C for 1 min. The mRNA levels were normalized to GAPDH and calculated using the comparative cycle threshold (ΔΔCt) method. The sequences of the primers are shown in Table 1.

**Table 1:** Sequences of the primers for qRT-PCR.

Gene	Primer Sequence
human C/Ebpα_F	5'-TGGACAAGAACAGCAACGAG-3'
human C/Ebpα_R	5'-CCATGGCCTTGACCAAGGAG-3'
human Glut4_F	5'-GGCCTCCGCAGGTTCTG-3'
human Glut4_R	5'-TTCGGAGCCTATCTGTTGGAA-3'
human Pparγ2_F	5'-AAATATCAGTGTGAATTACAGCAAACC-3'
human Pparγ2_R	5'-GGAATCGCTTTCTGGGTCAA-3'
human Fabp4_F	5'-GGTGGTGGAATGCGTCATG-3'
human Fabp4_R	5'-CAACGTCCCTTGGCTTATGC-3'
human Nox4_F	5'-AGCAGAACATTCCATATTACCTGTG-3'
human Nox4_R	5'-GATCCTCATCTCGGTATCTTGCT-3'
human Gapdh_F	5'-CGCTCTCTGCTCCTCCTGTT-3'
human Gapdh_R	5'-CATGGGTGGAATCATATTGG-3'
mouse Cd68_F	5'-CATCCCCACCTGTCTCTCTC-3'
mouse Cd68_R	5'-TTGCATTTCCACAGCAGAAG-3'
mouse Glucokinase_F	5'-GCTGGTACGACTTGTGCTG-3'
mouse Glucokinase_R	5'-TGGACACGCTTTCACAGG-3'
mouse Glut2_F	5'-TCAGAAGACAAGATCACCGGA-3'
mouse Glut2_R	5'-GCTGGTGTGACTGTAAGTGGG-3'
mouse Cd36_F	5'-ATGGGCTGTGATCGGAACTG-3'
mouse Cd36_R	5'-GTCTTCCCAATAAGCATGTCTCC-3'
mouse Srebp-1c_F	5'-ATCGCAAACAAGCTGACCTG-3'
mouse Srebp-1c_R	5'-AGATCCAGGTTTGAGGTGGG-3'
mouse Acc1_F	5'-ATGGGCGGAATGGTCTCTTTC-3'
mouse Acc1_R	5'-TGGGGACCTTGTCTTCATCAT-3'
mouse Cpt-1α_F	5'-TTGCCCTACAGCTCTGGCATTTCC-3'
mouse Cpt-1α_R	5'-GCACCCAGATGATTGGGATACTGT-3'
mouse Glut4_F	5'-TTGGAGAGAGAGCGTCCAAT-3'
mouse Glut4_R	5'-CTCAAAGAAGGCCACAAAGC-3'
mouse C/Ebpα_F	5'-AGGTGCTGGAGTTGACCAGT-3'
mouse C/Ebpα_R	5'-CAGCCTAGAGATCCAGCGAC-3'
mouse Pref-1_F	5'-TTCGGCCACAGCACCTATG-3'
mouse Pref-1_R	5'-GGGGCAGTTACACACTTGTCA-3'
mouse Hsl_F	5'-AGACACCAGCCAACGGATAC-3'
mouse Hsl_R	5'-ATCACCCTCGAAGAAGAGCA-3'
mouse Pparα_F	5'-TCAGGGTACCACTACGGAGTTCA-3'
mouse Pparα_R	5'-CCGAATAGTTCGCCGAAAGA-3'
mouse Nox4_F	5'-TGTCTGCATGGTGGTGGTATT-3'
mouse Nox4_R	5'-ACCTGAAACATGCAACAGCAG-3'
mouse Gapdh_F	5'-GTCTTCACTACCATGGAGAAGG-3'
mouse Gapdh_R	5'-TCATGGATGACCTTGGCCAG-3'

### 2.4 Western blotting (WB)

We lysed the cultured cells and homogenized tissues in RIPA buffer (Cat# P0045, Beyotime, China) containing a protease-inhibitor cocktail (Cat# 04693132001, Roche, Canada). After centrifugation at 14,000 rpm for 15 min at 4 °C, the protein concentrations were determined using a BCA protein assay kit (Cat# P0012, Beyotime, China). The cell and tissue lysates were then electrophoretically separated on 10% polyacrylamide gels and transferred onto PVDF membranes. The membranes were subsequently blocked with 5% nonfat dry milk dissolved in Tris-buffered saline/0.1% Tween 20 for 1 h at room temperature. After blocking, membranes were probed with the following diluted primary antibodies: anti-FASN (1:1000; Cell Signaling Technology, USA), anti-NOX4 (1:1000; Abcam, USA), anti-FABP4 (1:1000; Proteintech, China), anti-PPARγ (1:1000; Bioworld, USA), anti-β-actin (1:5000; Bioworld, USA) and anti-GAPDH (1:2000; Abcam, USA). After the membranes were incubated with the primary antibodies at 4 °C overnight, the membranes were incubated with goat anti-rabbit (1:1000, Beyotime, China) HRP-conjugated secondary antibodies and signals were detected using Image Lab software (Bio-Rad, USA).

### 2.5 Isolation and differentiation of primary white fat precursor cells

The primary white fat precursor cells were isolated from inguinal white adipose tissue (iWAT) of 4-week-old C57BL/6J male mice. The isolation method is consistent with that of mouse primary brown fat precursor cells our research group adopted [5]. For the differentiation of white fat precursor cells, the confluent cells were firstly induced with induction medium Ⅰ (DMEM/F12 containing 10% FBS, 0.5 mM isobutylmethylxanthine (IBMX) (Sigma), 1 μM dexamethasone (DEX) (Sigma), 860 nM insulin (Sigma), and 1 μM rosiglitazone (Sigma)) for 4 days (replaced every 2 days). After 4 days, induction medium Ⅰ was replaced with induction medium Ⅱ (DMEM/F12 supplemented with 10% FBS and 860 nM insulin (Sigma)) for 2 days and the precursor cells were differentiated into mature white fat cells. GKT137831 (S7171, Selleck) was added to the induction medium throughout the induction period. To further explore the effect of NOX4 on primary white fat precursor cell differentiation, small interfering RNA (siRNA) targeting mouse NOX4 gene sequence (CTCTTCATAGTTTGAGTAA) or mouse NOX4 overexpression plasmid was transfected into cells using Lipofectamine 2000 (Invitrogen, San Diego, CA) according to the manufacturer's instructions.

### 2.6 Human visceral preadipocytes (HPA-v) culture and differentiation

HPA-v (ScienCell Research Laboratories, USA) were cultured and induced to differentiate for the study of adipogenesis *in vitro* [25]. In detail, we maintained HPA-v cells in preadipocyte medium (PAM; ScienCell Research Laboratories) supplemented with 5% fetal bovine serum (FBS), 1% preadipocyte growth supplement (PAGS), and 1% penicillin/streptomycin solution at 37 °C in 5% CO_2_ until the cells achieved confluency. A differentiation medium (serum-free PAM supplemented with 5 μg/mL insulin, 1 μM DEX, 0.5 mM IBMX, and 1 μM rosiglitazone) was then employed to induce the differentiation of the cells for the first four days. The medium was then replaced with serum-free DMEM containing 5 μg/mL insulin and changed every three days until lipid droplets accumulation was observed. DHA (D7439, Sigma) or GKT137831 (S7171, Selleck) was added to the induction medium throughout the differentiation process. To explore the effect of the NOX4 gene on HPA-v differentiation, we mixed three siRNAs that targeted human NOX4 gene (GGACCCAATTCACTATCCA, CCAGGAGATTGTTGGATAA, and GCCGAACACTCTTGGCTTA) and transfected the siRNAs into HPA-v cells using Lipofectamine 2000 (Invitrogen, San Diego, CA) according to the manufacturer's instructions.

### 2.7 MTT and CCK8 assay

We employed the MTT (3-(4,5-dimethyl-2-thiazolyl)-2,5-diphenyl-2-H-tetrazolium bromide) and CCK8 (Cell Counting Kit 8) assay to evaluate the toxicity of DHA on HPA-v. For MTT assay, HPA-v was incubated with MTT (25 μg/mL) at 37 °C in 5% CO_2_ for 6 h an MTT detergent solution was added for 12 h at 37 °C. The optical density of the solution was then measured at 570 nm to evaluate cellular viability. Time course evaluation of DHA on HPA-v cell viability was examined 1, 4, 7, 10 days separately after adipocyte differentiation using a CCK8 (ApexBio, Houston, TX, USA). After incubation for 45 min at 37 °C, the absorbance was detected at 450 nm using a microplate reader Multiscan FC (Thermo Scientific, Waltham, MA, USA).

### 2.8 Oil red O staining

Oil red O staining was performed after the HPA-v cells differentiated into mature adipocytes. The cells were washed with phosphate-buffered saline (PBS) and stained with filtered oil red O (Sigma-Aldrich) solution (0.5% oil red O-isopropyl alcohol: H_2_O, 3:2, v/v) for 15 min at 37 °C. Following three washes with distilled water, the images of the cells were captured with an inverted microscope (Zeiss, Germany). To semi-quantify oil red O in positively stained cells, the stained cells were first washed with 60% isopropanol to remove the nonspecific stain, and the oil red O in lipid droplets was extracted with 100% isopropanol [26]. The absorbance value was determined using a microplate reader (Tecan GmbH, Grodig, Austria) at a wavelength of 510 nm.

### 2.9 Measurement of intracellular triacylglycerol (TG) content

The intracellular TG concentration was conducted using a TG assay kit (Applygen, Beijing, China) according to the manufacturer's instructions. Briefly, the cells were lysed with the lysis buffer provided in the TG assay kit. An appropriate amount of lysate was collected and heated at 70 °C for 10 min and then centrifuged at 2000 rpm for 5 min at room temperature. We then aspirated 10 μL of supernatant for the determination of TG concentrations using the prepared working solution provided in the kit. The remaining lysates were directly centrifuged at 14,000 rpm at 4 °C, and the supernatant was collected to determine the protein concentration using a BCA Protein Assay kit (Cat# P0012, Beyotime, China). The intracellular TG level was ultimately normalized with the protein concentration.

### 2.10 Determination of serum TG, total cholesterol (TC) and non-esterified fatty acids (NEFAs) concentrations

The serum TG and TC concentrations were conducted using TG and TC assay kits (Applygen, Beijing, China) according to the manufacturer's instructions. The detection steps of TG and TC were basically the same as the detection of intracellular TG. The serum NEFAs were conducted using assay kit (Cat# A042-2-1, Jiancheng, China) according to the manufacturer's instructions. Briefly, 4 μL sample, standard or double distilled water were incubated with 200 μL reagent 1 provided by the kit for 5 min at 37 °C, and the absorbance values were read at 546 nm and recorded. Then 50 μL reagent 2 was added to each well and incubated for another 5 min at 37 °C, the absorbance values were read at 546 nm and recorded. Finally, the concentration of NEFAs were calculated according to the formula provided in the manual.

### 2.11 Determination of serum insulin levels

After fasted for 6 h, whole blood was collected from the posterior vena cava of mice soon after euthanasia. Serum was then obtained after centrifuging the whole blood at 2000 rpm for 10 min at room temperature. Serum insulin levels were measured using an enzyme-linked immunosorbent assay (ELISA) kit (E-EL-M1382c, Elabscience, China) according to the manufacturer's instructions.

### 2.12 Histologic examination

Fresh adipose tissues were first soaked in 90% ethanol for 24 h and then transferred to 4% polyformaldehyde for fixation for another 24 h. The fixed tissues were dehydrated, embedded in paraffin, sectioned at 4-μm thickness, and then stained with hematoxylin-eosin (H&E). Tissue sections were examined, and photographs were taken under an Olympus BX51 (Olympus, Japan). The adipocyte diameter from the adipose tissue sections were analyzed by ImageJ software (v1.52a, National Institutes of Health, Bethesda, MD, USA).

### 2.13 UPLC−MS/MS-based targeted proteomics analysis of DHA in cultured HPA-v cells

HPA-v cells were differentiated and treated with DHA (20 μM) as described in section 2.6, and then the cells were washed with cold PBS three times, and scraped and centrifuged at 1000 rpm for 5 min. The cell pellets were resuspended in mammalian protein extraction reagent (Thermo Fisher Scientific) containing protease inhibitors and phosphatase inhibitors and lysed on ice for 30 min. After centrifugation at 18,000×*g* for 10 min, the cell lysates (150 μL, 3 μg/μL) were labeled with Paraformaldehyde-d 2 (CD_2_O), precipitated, digested and desalted by the same protocol as described previously [27]. A nanoACQUITY UPLC system coupled to a SYNAPT G2-Si mass spectrometer (Waters, Milford, MA, USA) was used for label-free quantification. The detailed UPLC−MS/MS conditions were the same as those described previously [27]. We searched the acquired data against the UniProt/SwissProt database (Homo sapiens, version 2018) using PEAKS Studio 8.5 (Bioinformatics Solutions Inc., Waterloo, Ontario, Canada). The search parameters were a precursor mass tolerance of 20 ppm and a fragment mass tolerance of 0.1 Da, and protein identifications with a false discovery rate (FDR) of less than 1% with at least one unique peptide were considered acceptable. Kyoto Encyclopedia of Genes and Genomes (KEGG)-pathway analysis was conducted by importing the proteins that displayed significant differential peptide abundance (i.e., a ratio higher than 1.50 or lower than 0.67, with a p value < 0.05) into Cytoscape and Database for Annotation, Visualization and Integrated Discovery (DAVID, v6.8).

### 2.14 Statistical analysis

All values are presented as the mean ± SEM and we executed statistical tests using GraphPad Prism 9. An unpaired Student's *t*-test was used for one-variable comparisons, and one- or two-way ANOVA was performed for two-variable comparisons. P < 0.05 was considered statistically significant.

## 3 Results

### 3.1 DHA improves obesity induced by a high-fat diet in mice

**Fig. 1:**
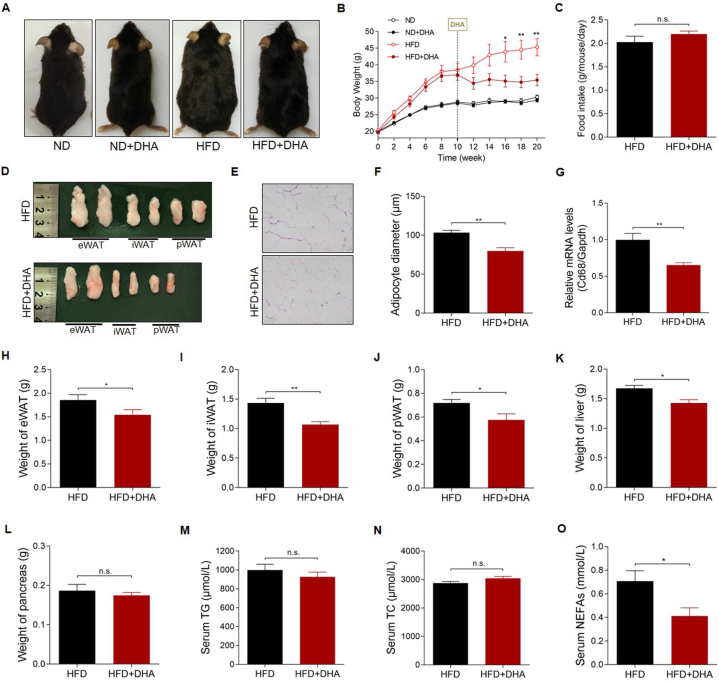
DHA improves a high-fat diet induced obesity in mice. (A) Representative photographs of ND- and HFD-fed mice treated with or without DHA. (B) BW of the experimental animals (n = 6–8 per group). (C) Food intake of the experimental animals (n = 6–8 per group). (D) Macroscopic view of representative sections of eWAT, iWAT, and pWAT from the experimental animals. (E–F) Representative H&E-stained images (200 × ) and adipocyte diameter of eWAT. (G) mRNA level of Cd68 gene in eWAT (n = 6–8 per group). (H–L) Weight of (H) eWAT, (I) iWAT, (J) pWAT, (K) liver and (L) pancreas of the experimental animals (n = 6–8 per group). (M–O) Serum TG, TC and NEFAs levels of the experimental animals (n = 6–8 per group). The values are mean ± SEM. *P < 0.05 and **P < 0.01; n.s., not significant (p > 0.05).

To confirm the potential therapeutic effect of DHA on obesity, we first used a high-fat diet to induce obesity for 10 weeks, and then employed DHA to treat the obese mice orally (25 mg/kg/day) for another 10 weeks. Our results showed that the DHA-treated obese mice became significantly thinner, whereas the DHA-treated ND-fed mice were not significantly different (Fig. 1A). DHA maintained or even reduced the BW of obese mice, with the weight loss of obese mice reaching 21.8% (Fig. 1B). Consistent with the BW change, DHA-treated HFD mice possessed a much lower volume and weight of epididymal white adipose tissue (eWAT), iWAT, and perirenal white adipose tissue (pWAT) (P < 0.05) (Fig. 1D, H-J). H&E staining of eWAT also revealed that the adipocyte size was much smaller in DHA-treated obese mice than in control mice (Fig. 1E and F). Meanwhile, the expression of macrophage marker gene cluster of differentiation 68 (Cd68) was significantly decreased in eWAT of HFD-fed mice treated with DHA (Fig. 1G). Along with the decreased weight of adipose tissues, the weight of liver also decreased significantly while pancreas weight did not change significantly under the intervention of DHA (Fig. 1K and L). DHA significantly decreased serum NEFAs level, but had no significant effect on serum TG and TC levels (Fig. 1M, N and O). We observed no significant difference in the food intake of obese mice treated with or without DHA (Fig. 1C), indicating that the weight loss is not due to appetite decreases. These results indicated that DHA significantly improved obesity in DIO mice.

### 3.2 The improvement of DHA on glucose metabolism depends on weight loss

**Fig. 2:**
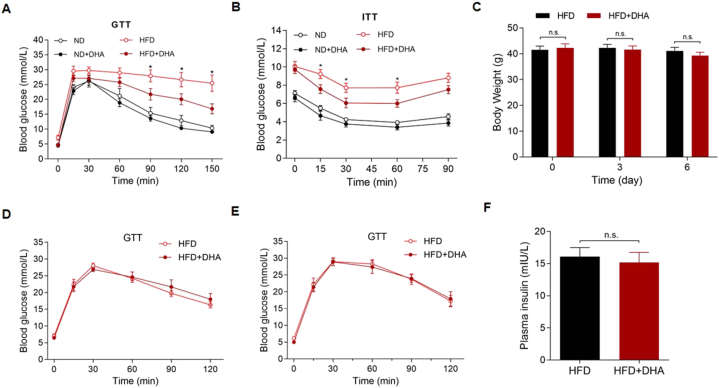
Improvement in glucose metabolism in mice depends upon weight loss. (A) GTT results of the ND- and HFD-fed mice treated with or without DHA for 10 weeks (n = 6–8 per group). (B) ITT results of the ND- and HFD-fed mice treated with or without DHA for 10 weeks (n = 6–8 per group). (C) BW of DIO mice treated with or without DHA for three and six days (n = 8 per group). (D–E) GTT results of DIO mice treated with or without DHA for (D) three days and (E) six days (n = 8 per group). (F) Serum insulin levels of DIO mice treated with or without DHA for six days (n = 8 per group). The values are mean ± SEM. *P < 0.05; n.s., not significant (p > 0.05).

We performed GTT and ITT to assess the effects of DHA on glucose homeostasis and insulin resistance. GTT results showed that long-term (10-week) treatment of DHA-treated obese mice with DHA induced a faster diminution in blood glucose concentration upon glucose injection compared to control animals (Fig. 2A). The ITT results also showed better insulin sensitivity in the long-term DHA-treated obese mice than in the control mice (Fig. 2B). It has been previously reported that artemisinin and its derivatives can induce the conversion of α cells to functional β-like cells, thereby increasing the secretion of insulin [28]. Therefore, in order to explore whether the improvement in glucose metabolism in obese mice was due to a change in islet cell function or to a decline in body weight, we treated the DIO mice showing an average BW of about 42 g for relatively short terms of three and six days. During treatment, the BW did not change significantly (P > 0.05) (Fig. 2C), and the GTT results showed that the efficiency of glucose metabolism was not significantly improved in obese mice treated with DHA for either three or six days (P > 0.05) (Fig. 2D and E). In addition, we did not detect a significant change of serum insulin levels in obese mice treated with DHA for six days (P > 0.05) (Fig. 2F). These results suggested that the effects of DHA on glucose metabolism were secondary to the reduction in mouse body weight.

### 3.3 DHA inhibits adipocyte differentiation and the expression of NOX4 in WAT of mice

**Fig. 3:**
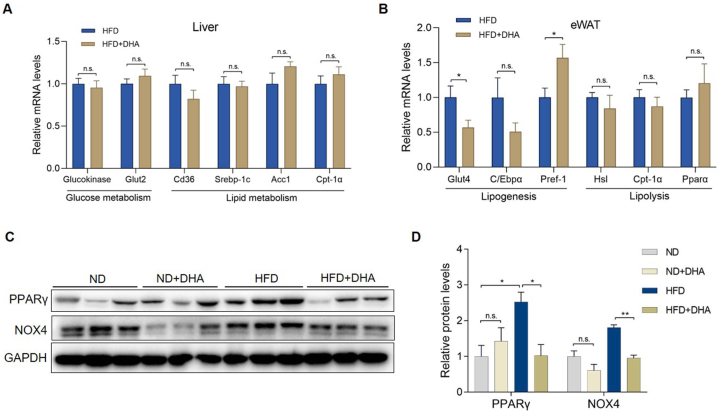
DHA inhibits the expression of adipose differentiation-related genes in WAT of mice fed with HFD. (A) mRNA levels of glucose metabolism-related genes (Glucokinase and Glut2) and lipid metabolism-related genes (Cd36, Srebp-1c, Acc1 and Cpt-1α) in the liver of HFD-fed mice treated with or without DHA (n = 6–8 per group). (B) mRNA levels of lipogenesis-related genes (Glut4, C/Ebpα and Pref-1) and lipolysis-related genes (Hsl, Cpt-1α and Pparα) in the eWAT of HFD-fed mice treated with or without DHA (n = 6–8 per group). (C) Representative western blots of PPARγ and NOX4 in the eWAT of ND- and HFD-fed mice treated with or without DHA, and (D) corresponding quantified signal intensities (n = 3 per group). The values are mean ± SEM. *P < 0.05 and **P < 0.01; n.s., not significant (p > 0.05).

The weight reduction of WAT and liver plays a major role in the weight reduction of mice fed with HFD, so we next analyzed transcription level of the metabolism related genes in the WAT and liver. We did not observe significant changes in mRNA levels of liver glucose metabolism-related genes such as Glucokinase and Glut2 and lipid metabolism-related genes such as cluster of differentiation 36 (Cd36), Sterol Regulatory element binding protein-1c (Srebp-1c), acetyl-CoA carboxylase1 (Acc1) and carnitine palmitoyltransferase 1a (Cpt-1α) (Fig. 3A). Then we detected the expression of genes related to adipocyte differentiation such as glucose transporter 4 (Glut4), C/Ebpα, Pparγ and preadipocyte factor-1 (Pref-1) in the eWAT of mice fed with HFD, and the results showed that DHA significantly down-regulated the expression of differentiation promoting genes Glut4 and Pparγ at mRNA or protein level, and C/Ebpα gene also showed a downward trend upon DHA treatment (Fig. 3B, C and D). Pref-1, an early negative regulator of adipogenic differentiation [29], was significantly upregulated after DHA treatment (Fig. 3B). The lipolysis related-genes such as hormone-sensitive lipase (Hsl), Cpt-1α and peroxisome proliferator-activated receptor α (Pparα) in the eWAT of HFD-fed mice were not significantly changed under the intervention of DHA (Fig. 3B). In addition, we found that the protein level of NOX4 in adipocytes of eWAT was significantly downregulated after DHA treatment (P < 0.05) (Fig. 3C and D). These results indicating that DHA inhibited the differentiation of white adipocytes *in vivo*.

### 3.4 DHA inhibits the differentiation of white adipose precursor cells *in vitro*

**Fig. 4:**
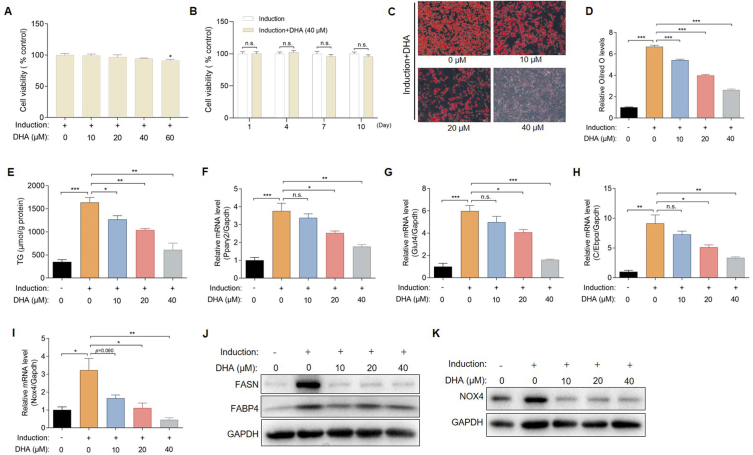
DHA suppresses the adipocyte differentiation *in vitro*. (A) Cell viability of DHA-treated HPA-v cells determined by MTT. (B) Cell viability of DHA-treated HPA-v cells during the whole differentiation process by CCK8. (C) Representative oil red O-stained images (100 × ) of HPA-v cells treated with DHA during differentiation induction (n = 3 per group). (D–E) (D) Semiquantitative analysis of oil red O levels and (E) TG levels in HPA-v cells treated with DHA during differentiation induction (n = 4 per group). (F–H) mRNA levels of adipocyte differentiation-related genes (Pparγ2, Glut4 and C/Ebpα) in HPA-v cells treated with DHA during differentiation induction (n = 4 per group). (I) Nox4 mRNA levels in HPA-v cells treated with DHA during differentiation induction (n = 4 per group). (J) FASN and FABP4 protein levels in HPA-v cells treated with DHA during differentiation induction. (K) Western blots of NOX4 in HPA-v cells treated with DHA during differentiation induction. The values are mean ± SEM. *P < 0.05, **P < 0.01, and ***P < 0.001; n.s., not significant (p > 0.05). (For interpretation of the references to colour in this figure legend, the reader is referred to the Web version of this article.)

Next, we assessed whether DHA inhibited fat formation by exploiting a well-characterized model of inducing adipose precursor HPA-v cells into typical white adipocytes. As MTT assay showed that DHA at concentrations ranging from 10 to 40 μM posed no cellular toxicity in HPA-v cells (Fig. 4A), DHA less than 40 μM was employed *in vitro.* After the cultured HPA-v cells attained confluency, they were induced to differentiate with or without DHA treatment. During the whole differentiation process of HPA-v cells, 40 μM DHA treatment had no significant effect on cell viability (Fig. 4B). After the induction was completed, oil red O-stained images and corresponding semi-quantification results depicted a DHA-dependent attenuation of lipid-droplet accumulation in adipocytes (P < 0.05) (Fig. 4C and D). Analysis of the TG content in the different groups of adipocytes also confirmed that DHA significantly curtailed lipid accumulation during adipocyte differentiation (Fig. 4E).

We next examined the expression of typical adipocyte differentiation-related genes, and demonstrated that—consistent with cellular phenotype—the mRNA expression levels of the adipogenesis markers Pparγ2, Glut4 and C/Ebpα were significantly inhibited by DHA in a dose-dependent manner (P < 0.05) (Fig. 4F–H). Western blotting results showed that the expression of FASN, a key enzyme in adipogenesis, and FABP4, an important marker of adipogenesis, were both significantly inhibited by DHA (Fig. 4J). To further examine whether the change in NOX4 expression in adipose tissue was a direct effect of DHA, we assessed the effect of DHA on the expression of NOX4 in cultured HPA-v cells. Consistently, the qRT-PCR and WB data showed that DHA significantly downregulated the expression of NOX4 at both the mRNA and protein levels in HPA-v cells compared with the induction group without DHA treatment (Fig. 4I, K). Collectively, these data suggested that DHA inhibited the differentiation of adipocytes.

### 3.5 Pharmacological inhibition of NOX4 inhibits the differentiation of adipose precursor cells *in vitro*

**Fig. 5:**
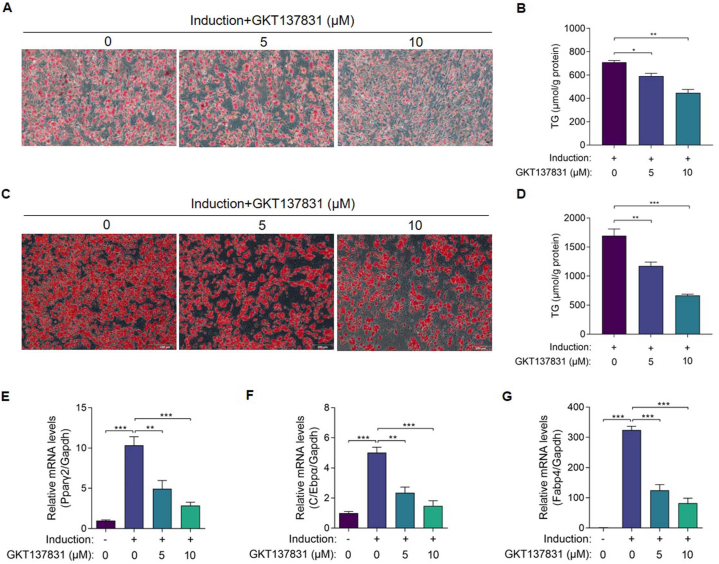
Pharmacological inhibition of NOX4 inhibits the differentiation of adipose precursor cells. (A and B) (A) Representative oil red O-stained images (200 × ) and (B) TG levels of differentiated mouse primary white fat precursor cells treated with GKT137831 (n = 3 per group). (C and D) (C) Representative oil red O-stained images (100 × ) and (D) TG levels of differentiated HPA-v cells treated with GKT137831 (n = 3 per group). (E–G) mRNA levels of adipocyte differentiation-related genes (Pparγ2, C/Ebpα, and Fabp4) in HPA-v cells treated with GKT137831 (n = 3 per group). The values are mean ± SEM. *P < 0.05, **P < 0.01 and ***P < 0.001. (For interpretation of the references to colour in this figure legend, the reader is referred to the Web version of this article.)

In order to investigate whether NOX4 plays a role in DHA's adipocyte differentiation inhibition effect, NOX4 inhibitor GKT137831 was used to observe its effect on adipocyte differentiation. The oil red O staining images and quantitative TG results showed that GKT137831 significantly inhibited lipid accumulation in both differentiation-induced mouse primary white fat precursor cells (Fig. 5A and B) and HPA-v cells (Fig. 5C and D). Similarly, the transcriptional level of the adipocyte differentiation-related genes Pparγ2, C/Ebpα, and Fabp4 in HPA-v cells were downregulated in a drug concentration-dependent manner (Fig. 5E–G).

### 3.6 NOX4 plays a role in DHA inhibiting adipocyte differentiation

**Fig. 6:**
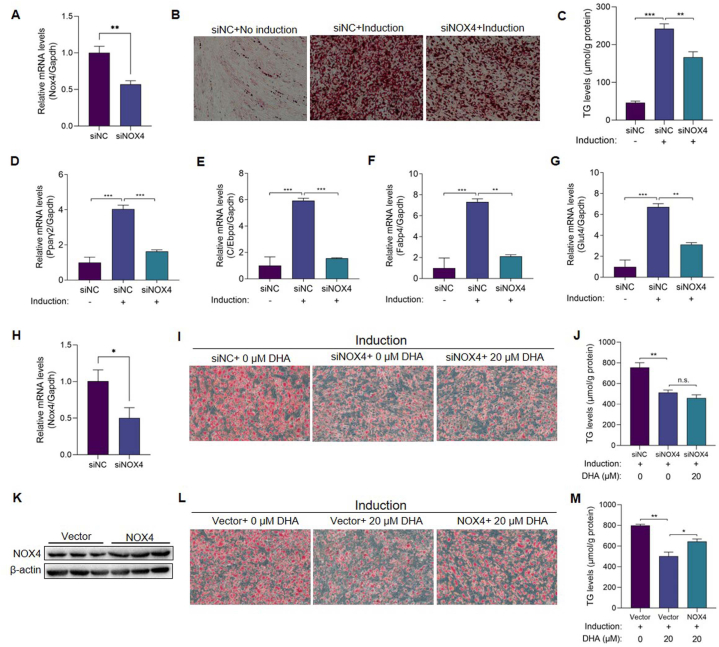
NOX4 plays a role in DHA's adipocyte differentiation inhibition effect. (A) The knocking down efficiency of the Nox4 siRNA was assessed by qRT-PCR in HPA-v cells at 24 h after transfection (n = 3 per group). (B–C) (B) Representative oil red O-stained images (200 × ) and (C) TG levels of differentiated HPA-v cells transfected with NOX4 siRNA (n = 3 per group). (D–G) mRNA levels of adipocyte differentiation-related genes (Pparγ2, C/Ebpα, Fabp4 and Glut4) in HPA-v cells transfected with NOX4 siRNA (n = 3 per group). (H) The transcription level of Nox4 gene was assessed by qRT-PCR in primary white fat precursor cells 24 h after Nox4 siRNA transfection (n = 3 per group). (I–J) (I) Representative oil red O-stained images (200 × ) and (J) TG levels of DHA-treated differentiated primary white fat precursor cells transfected with Nox4 siRNA (n = 3 per group). (K) The NOX4 overexpression efficiency in primary white fat precursor cells was assessed by WB (n = 3 per group). (L–M) (L) Representative oil red O-stained images (200 × ) and (M) TG levels of DHA-treated differentiated primary white fat precursor cells transfected with Nox4 overexpression plasmid. The values are mean ± SEM. *P < 0.05, **P < 0.01 and ***P < 0.001; n.s., not significant (p > 0.05). (For interpretation of the references to colour in this figure legend, the reader is referred to the Web version of this article.)

We then investigated whether NOX4 plays a role in DHA inhibiting adipocyte differentiation. Firstly, we knocked down NOX4 with siRNAs in HPA-v cells, and qRT-PCR results showed that the NOX4 gene in HPA-v cells was knocked down nearly 50% at 24 h after siRNA transfection (P < 0.01) (Fig. 6A). At the end of the induction, oil red O staining showed that the density of lipid droplets in the NOX4-knockdown group was significantly lower than that in the control group, and the change in intracellular TG levels was consistent with the results of oil red O staining (P < 0.01) (Fig. 6B and C). Consistent with cellular phenotype, the qRT-PCR results revealed that the adipocyte differentiation-related genes Pparγ2, C/Ebpα, Fabp4 and Glut4 were significantly downregulated upon NOX4 knocking down (P < 0.01) (Fig. 6D–G), indicating that the NOX4 gene was critical in promoting adipocyte differentiation. In addition, we also knocked down Nox4 gene in mouse primary white fat precursor cells using siRNA (Fig. 6H). The oil red O staining and TG levels showed that knocking down Nox4 inhibited adipogenic differentiation of preadipocytes, but its inhibition was not further aggravated by 20 μM DHA treatment (Fig. 6I and J). We then over-expressed Nox4 gene in primary white fat precursor cells (Fig. 6K), the oil red O staining and TG levels showed that the over-expression of Nox4 partially reversed the inhibition effect of DHA on preadipocytes differentiation (Fig. 6L and M). These results affirmed that NOX4 accounted for the differentiation of adipocytes, and that the inhibition of NOX4 by DHA was the key underlying mechanism by which DHA inhibited adipose differentiation.

### 3.7 UPLC–MS/MS-based targetomic analysis can be used to explore the effects of DHA on adipocytes

**Fig. 7:**
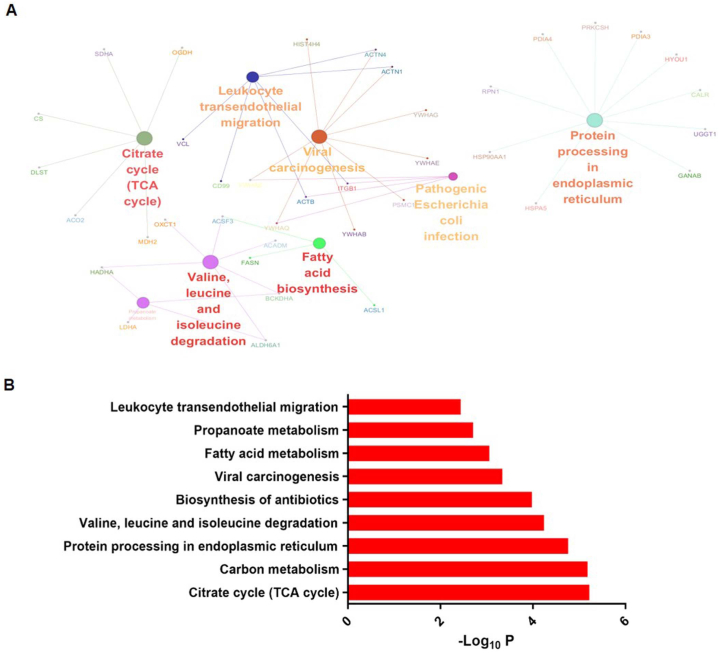
KEGG-pathway analysis of conformationally modified proteins in DHA-treated, differentiating adipocytes identified by targeted proteomics. (A) KEGG pathway enrichment analysis of 85 conformationally altered proteins was performed with the ClueGO app included in Cytoscape. Pathways with a p value < 0.01 (by two-sided hypergeometric test) and the inclusion of at least three genes are summarized. (B) Gene Ontology analysis of KEGG pathways using Database for Annotation, Visualization and Integrated Discovery (DAVID, v6.8).

In order to assess the effect of DHA on adipocytes more comprehensively, we used a target-responsive accessibility-profiling approach that measures DHA-induced steric hindrance of proteins via UPLC–MS/MS-based global-profiling of accessibility changes in reactive lysines. This method was previously termed as targetome [27]. During the differentiation of HPA-v cells, we added 20 μM DHA to the culture medium, and the cells were harvested until completion of induction. The DHA targetome was thus profiled, and we discerned that DHA treatment altered the conformations of 85 proteins (Data S1). Of these, FASN (one of the key enzymes in the fatty acid synthetic pathway), was shown to be conformationally changed upon DHA treatment, with reduced abundance of two unique dimethylated K-containing peptides (Data S1). KEGG-pathway analysis showed that the modified proteins were significantly enriched in fatty acid metabolism; TCA cycle; carbon metabolism; valine, leucine, and isoleucine degradation; and protein processing in endoplasmic reticulum (Fig. 7A and B). These data indicated that DHA significantly affected the metabolism of adipocytes.

## 4 Discussions

Obesity and its related disorders have developed into a global pandemic, and although the U.S. Food and Drug Administration (FDA) has approved five drugs for long-term weight management, their modest efficacy and unfavorable side effects limit their clinical application [30]. As a clinical antimalarial drug, artemisinin and its derivatives possess the advantages of moderate pricing, little toxicity, and side-effects. Recent studies have shown that artemisinin derivatives improved HFD-induced obesity by promoting the browning of white fat and enhancing brown adipose tissue function [23]. However, in a follow-up report these authors did not achieve a similar effect, and no typical browning-related genes (such as Ucp1, Pgc-1α, or Prdm16) were found among the differentially expressed genes identified by RNA-Seq [31]. A recent report showed that artemisinin derivatives DHA or artesunate (ATS) inhibited adipogenic differentiation of preadipocytes 3T3-L1 by up-regulating C/Ebp-Homologous Protein (CHOP) [31]. In addition, some studies have shown that artemisinin derivatives can inhibit the differentiation of preadipocytes through reducing expression of C/Ebp δ [32] or reducing the expression and/or phosphorylation levels of C/Ebp-α, PPAR-γ, FAS, perilipin A, and STAT-3 [33]. Therefore, the mechanism(s) underlying the anti-obesity effects of DHA remains controversial. In the present study we demonstrated that DHA improved HFD-induced obesity and enhanced glucose metabolism in obese mice. DHA treatment also reduced the white fat mass, possibly by inhibiting adipocyte differentiation. Our research confirms for the first time that the reduction in NOX4 in adipocytes served as a molecular mechanism mediating the anti-obesity effects of DHA.

White adipose tissue stores energy through adipogenesis in response to caloric excess, but excessive WAT poses a serious threat to metabolic health [34]. The number of adipocytes in lean and obese adults is in a dynamic and stable state, adipocyte renewal and lipid accumulation constitute important events in adipose tissue enlargement [12], and we acknowledge that adipocytes are generated through adipogenesis from specific precursor cells. In our study, DHA treatment reduced the volume of white fat and the expression of Pparγ in the eWAT in DIO mice, suggesting that DHA inhibited the differentiation of adipocytes; and this was confirmed by the adipogenic differentiation model of HPA-v cells *in vitro*. The inhibition of adipogenesis may thus comprise the principal cause of the anti-obesity effect of DHA. It is noteworthy that the decrease of liver weight and serum NEFAs in obese mice treated with DHA we observed in our study could be secondary to the change of fat tissue metabolism or through other mechanisms, which needs further study.

It is well known that aerobic cells produce a series of ROS in metabolic processes; these include O_2_^−^, H_2_O_2_, HO_2_ • and • OH [35]; and free radicals in the body regulate the balance of cellular life and death through changes in their concentrations. In addition to their functions in apoptosis and necrosis, the broader physiologic significance of low ROS concentrations reflects the activation of transcription factors and the promotion of cellular proliferation and differentiation [[36], [37], [38]]. ROS are thought to interact with several pathways that affect the transcriptional machinery required for mesenchymal stem cells differentiation, and tightly regulated levels of ROS are therefore critical for the terminal differentiation of mesenchymal stem cell [17].

Among various enzymes responsible for ROS generation, NOXs are the major and most widely studied sources of ROS [16,39]; and NOX4 is an important source of ROS production in adipocytes, playing a crucial role in adipogenesis [22,40]. Previous studies revealed that overexpression of NOX4 in human preadipocytes promoted the accumulation of fat droplets, while NOX4 knockdown inhibited adipocyte differentiation [17,22]. Both our *in vivo* and *in vitro* experiments showed that DHA was able to significantly downregulate NOX4 expression in the WAT of obese mice, and that the adipose precursor cells induced to differentiate after inhibition of NOX4 by siRNA or GKT137831 also reduced the differentiation of adipocytes. And overexpressed NOX4 in DHA-treated cells significantly weakened the inhibitory effect of DHA. In terms of a subserving mechanism of action, a previous report showed that H_2_O_2_ induced activation of cAMP response element-binding protein (CREB) transcription [17]. CREB has been implicated as an early regulator of the adipocyte differentiation program, and in 3T3-L1 preadipocytes, CREB was identified as a transcription factor that regulated the adipocyte marker C/Ebpβ during adipocyte differentiation [41,42]. In addition to CREB, other investigators reported that preadipocytes released the EGF-like protein Pref-1 to maintain the undifferentiated state of preadipocytes. Pref-1 activates the MEK/ERK-pathway to block the induction of the key transcription factor Pparγ2 for further differentiation [43,44]. Pref-1 expression is completely lost in the course of adipocyte differentiation, but the downregulation of Pref-1 was prevented by Nox4 knockdown using a Nox4 siRNA [22]. These studies revealed that the expression of NOX4 was closely related to abnormal cellular metabolic pathways, and our data also showed that inhibition of NOX4 downregulated the expression of adipocyte differentiation related genes such as Pparγ2 and C/Ebpα. It is hypothesized that the regulation of NOX4 expression by DHA may be part of the molecular mechanism by which DHA inhibits adipose differentiation.

In order to assess the effect of DHA on adipocytes more comprehensively, we implemented UPLC–MS/MS-based targetome analysis in DHA-treated HPA-v cells. The targetomics approach of labeling the protein with CD_2_O before digestion has been proven to directly detect alterations in potential target proteins by quantifying unique peptides with and without treatment [27,45]. We can therefore define a group of peptides with altered abundances and thus reveal potential DHA-protein interactions. In our experimental setting, a total of 104 peptides mapped to 85 proteins were found to be conformationally changed after DHA treatment. By KEGG analysis, we observed that these proteins were significantly enriched in endogenous metabolism pathways, such as fatty acid metabolism; TCA cycle; carbon metabolism; valine, leucine, and isoleucine degradation; and protein processing in endoplasmic reticulum pathways. Based upon published studies, there is an elevated flux within the TCA cycle and fatty acid oxidation during adipocyte differentiation and enlargement [46]. It has been reported that catabolism of branched-chain amino acids, such as leucine and isoleucine fueled adipocyte differentiation and lipogenesis [47]; and endoplasmic reticulum stress was also associated with adipocyte differentiation [48]. FASN, which plays a key role in reducing lipid accumulation in adipocytes, was additionally specifically demonstrated to be conformationally changed upon DHA treatment. Thus, the targetomics analysis provided additional evidence that DHA affected adipocyte metabolism.

There are limitations of our study. Firstly, we did not use white adipose-specific NOX4 overexpression mice to verify the key role of NOX4 in DHA inhibiting adipocyte differentiation. Secondly, we did not verify the effect of GKT137831 on body weight and lipid metabolism of WAT of DIO mice *in vivo*. These limitations deserve our in-depth research in the future.

## 5 Conclusions

In summary, our study revealed that DHA exerts a therapeutic effect on obesity in an HFD-induced obese mice by inhibiting adipocyte differentiation, and that its mechanism of action may lie in its inhibitory effect on the ROS-producing enzyme NOX4 and abnormal cell-metabolism pathways. The study of these functions and underlying molecular mechanisms provides a greater theoretical basis for DHA in the treatment of obesity, enabling the potential for DHA to be applied as a therapy for obese patients.

## Consent for publication

All authors approved the final version of the manuscript for publication.

## Availability of data and materials

Raw data from our UPLC–MS/MS-based targetomics analysis have been deposited into the iProX system with the project number IPX0003263000, and can be viewed at https://www.iprox.cn/page/PSV023.html;?url=1626145160861VLpW by entering the accession number (password) “RahH”. The other raw data supporting the conclusions of this article will be made available by the authors, upon reasonable request and without undue reservation.

## Ethics approval

This animal study was reviewed and approved by the Institutional Animal Care and Use Committee of Nanjing Medical University.

## Author contribution statement

Hu Hua, Mengqiu Wu: Performed the experiments; Analyzed and interpreted the data; Wrote the paper.

Tong Wu: Analyzed and interpreted the data; Wrote the paper.

Yong Ji, Lv Jin, Yang Du, Yue Zhang, Songming Huang, Aihua Zhang: Contributed reagents, materials, analysis tools or data.

Guixia Ding, Qianqi Liu: Conceived and designed the experiments.

Zhanjun Jia: Conceived and designed the experiments; Analyzed and interpreted the data; Wrote the paper.

## Funding statement

The study was supported by grants from the 10.13039/501100001809National Natural Science Foundation of China (82090020, 82090022, 82000642), the 10.13039/501100004608Natural Science Foundation of Jiangsu Province (BK20191123), and the Science and Technology Development Foundation of 10.13039/501100007289Nanjing Medical University (NMUB2020038).

## Data availability statement

Data associated with this study has been deposited at Raw data from our UPLC–MS/MS-based targetomics analysis have been deposited into the iProX system with the project number IPX0003263000, and can be viewed at https://www.iprox.cn/page/PSV023.html;?url=1626145160861VLpW by entering the accession number (password) “RahH”.

## Declaration of interest’s statement

The authors declare no competing interests.
