# Supplementary material for: Reduction of NADPH oxidase 4 in adipocytes contributes to the anti-obesity effect of dihydroartemisinin
Source: Heliyon. 2023 Feb 25;9(3):e14028. doi: 10.1016/j.heliyon.2023.e14028 (PMC10006843; doi:10.1016/j.heliyon.2023.e14028)

PPARγ for Figure 3C

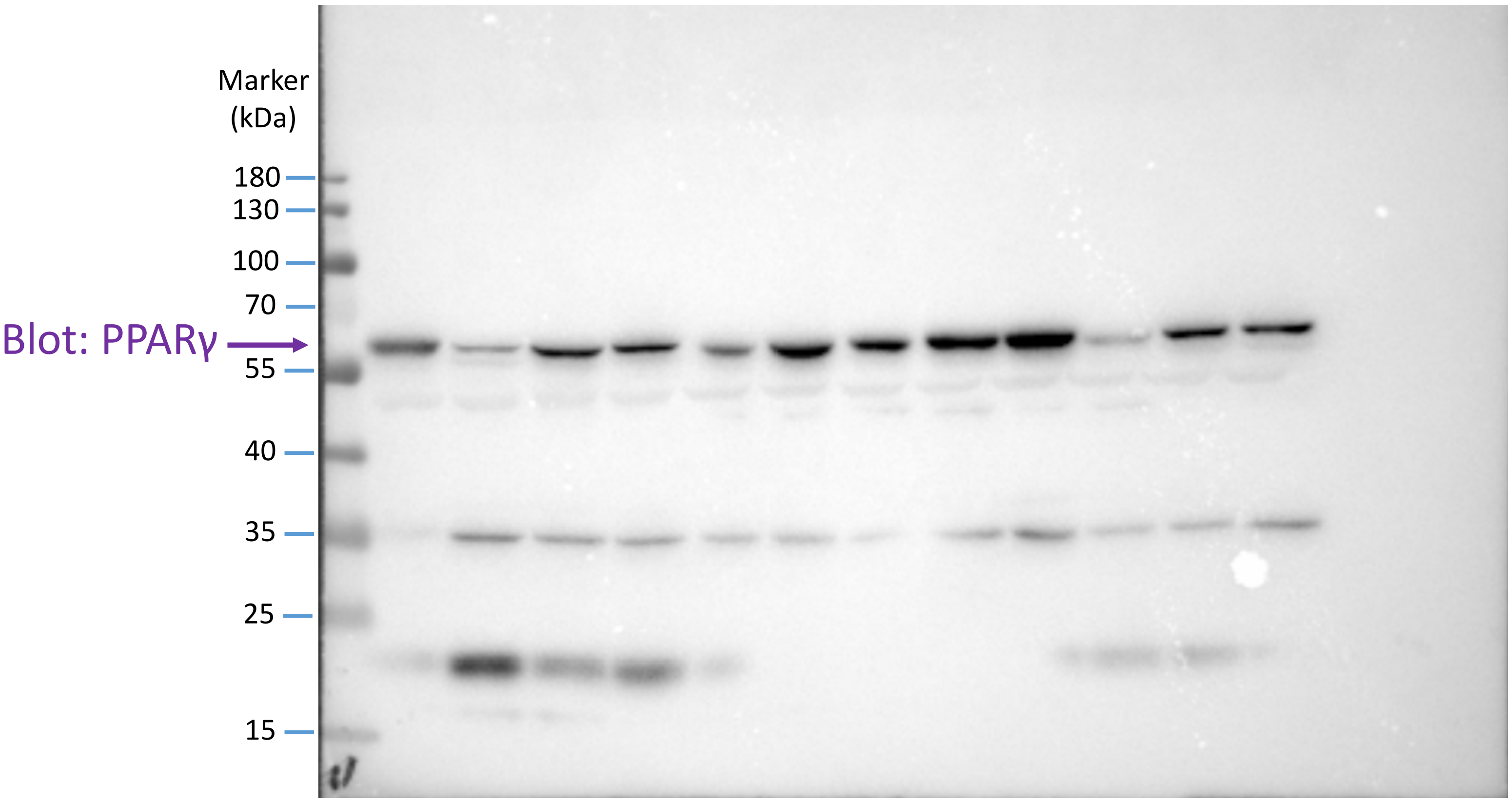

NOX4 for Figure 3C

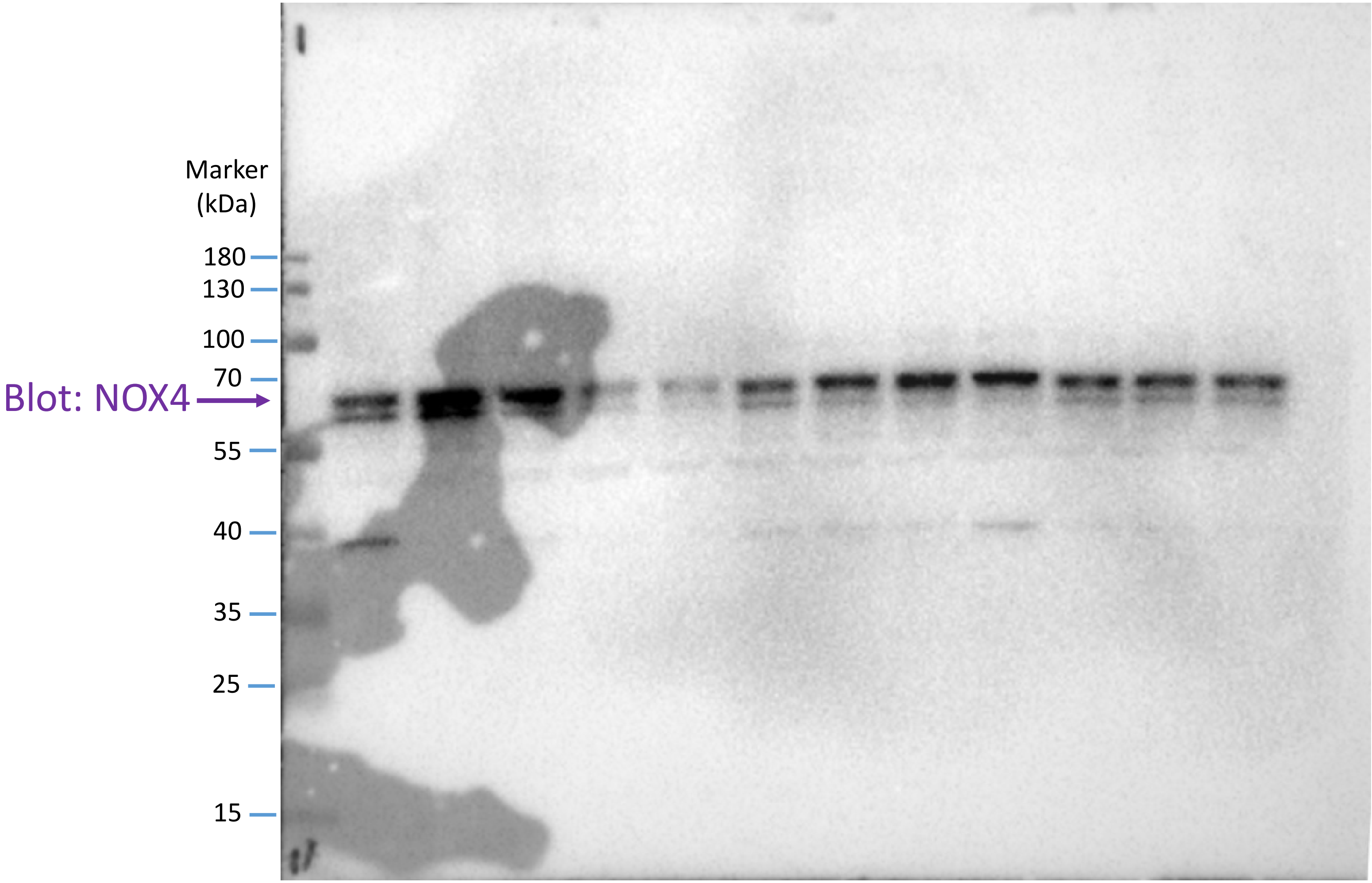

# GAPDH for Figure 3C

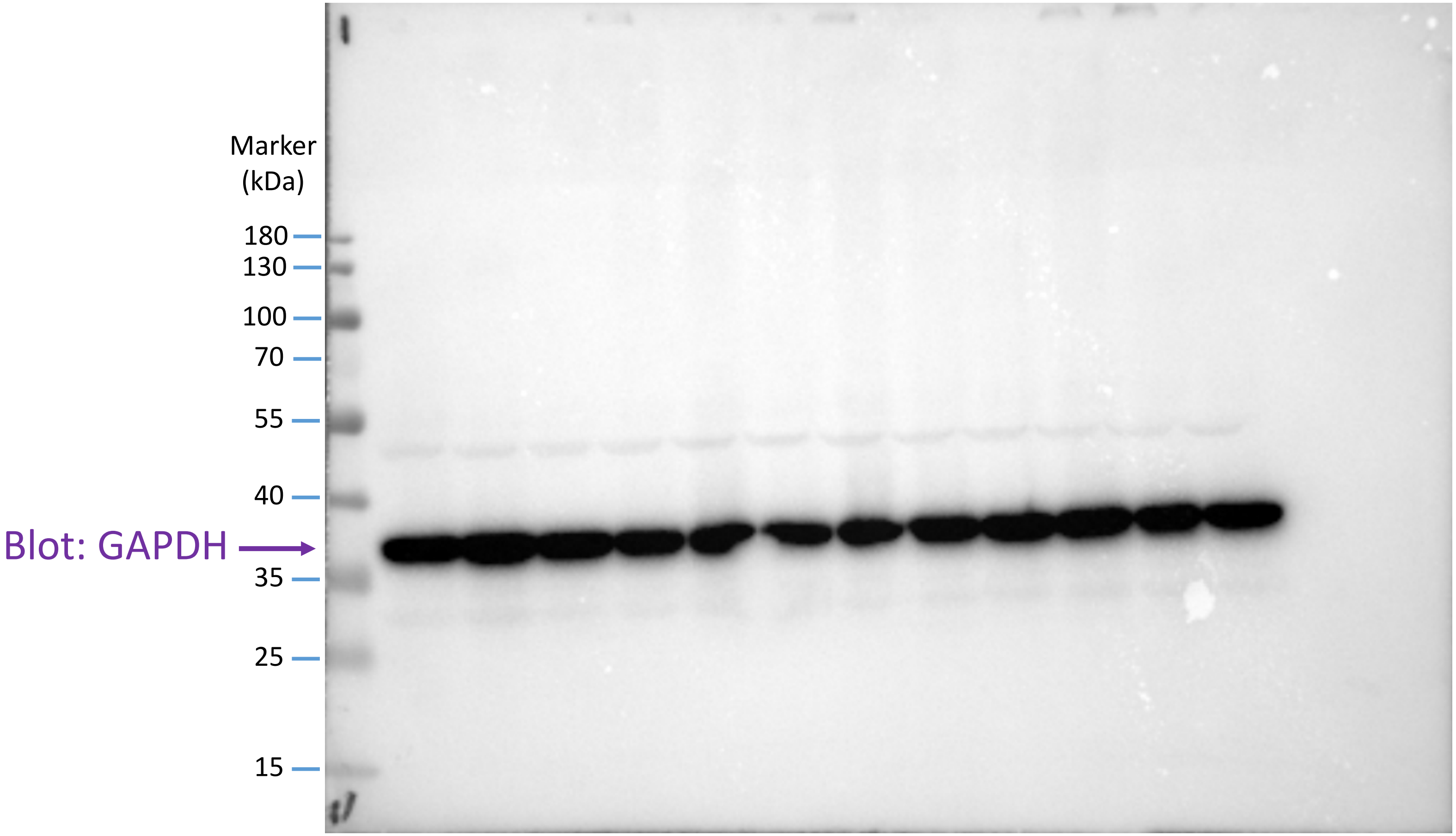

FASN for Figure 4J

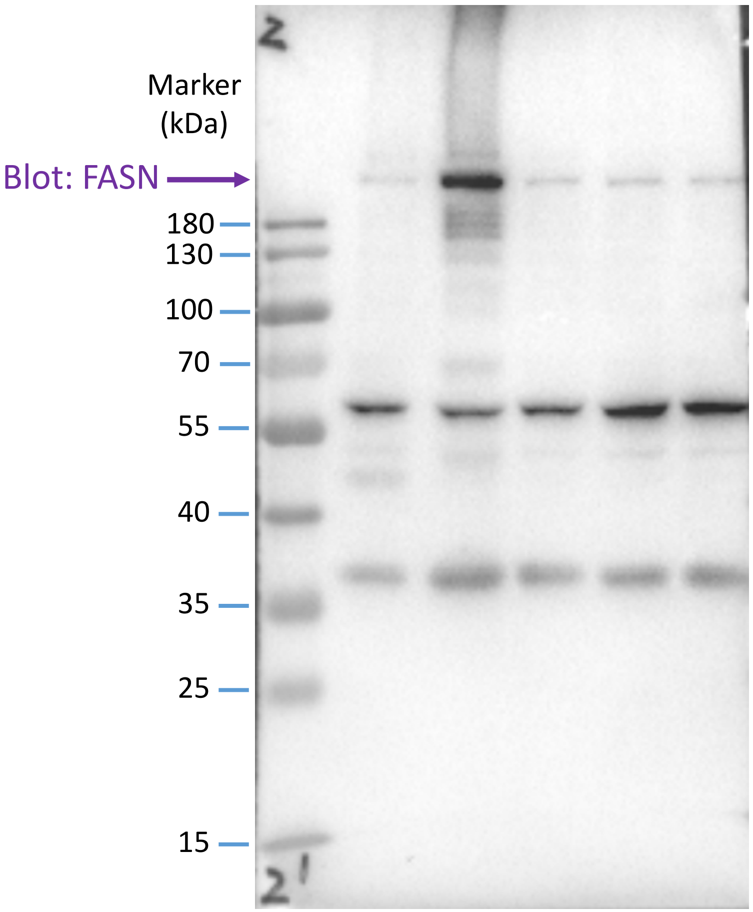

GAPDH for Figure 4J

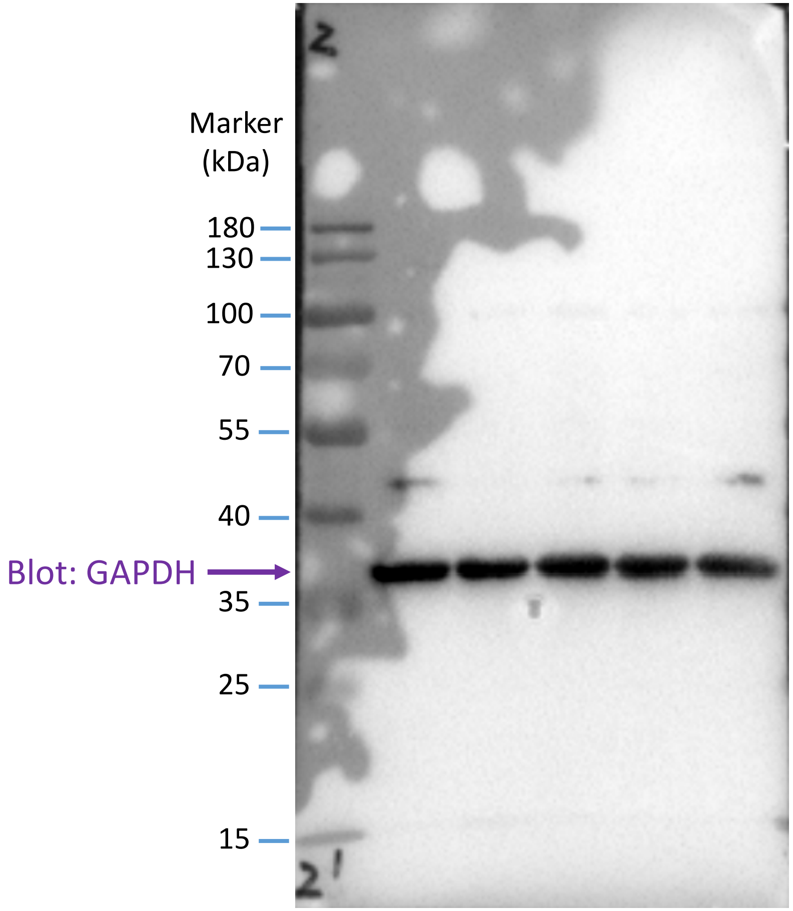

FABP4 for Figure 4J

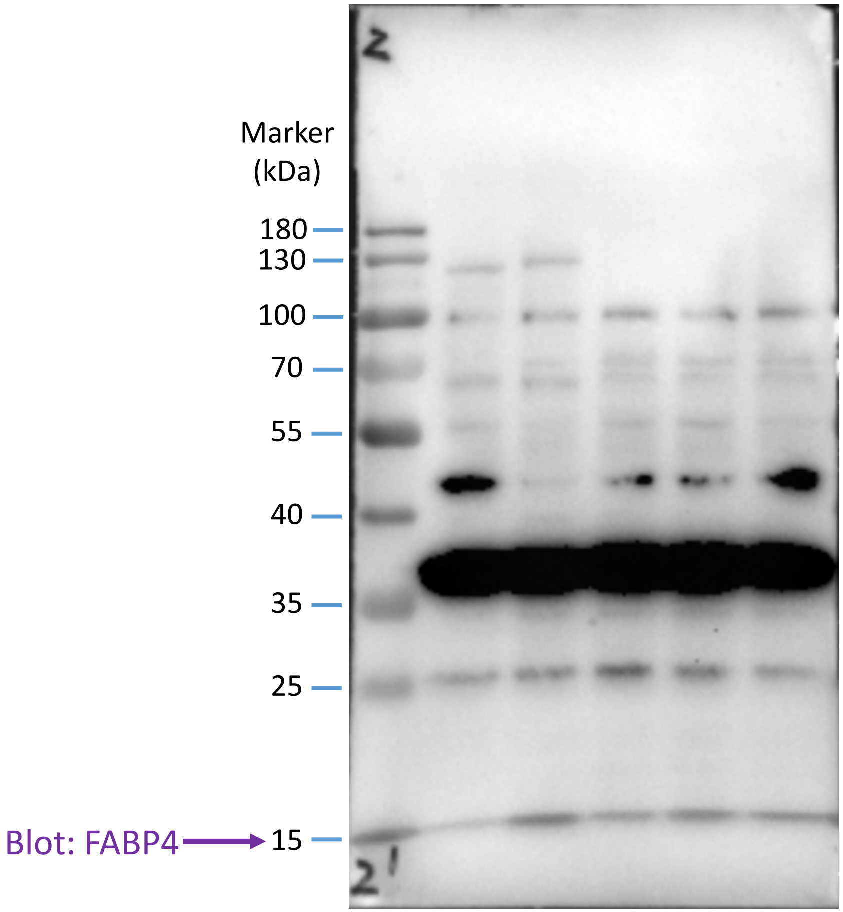

NOX4 for Figure 4K

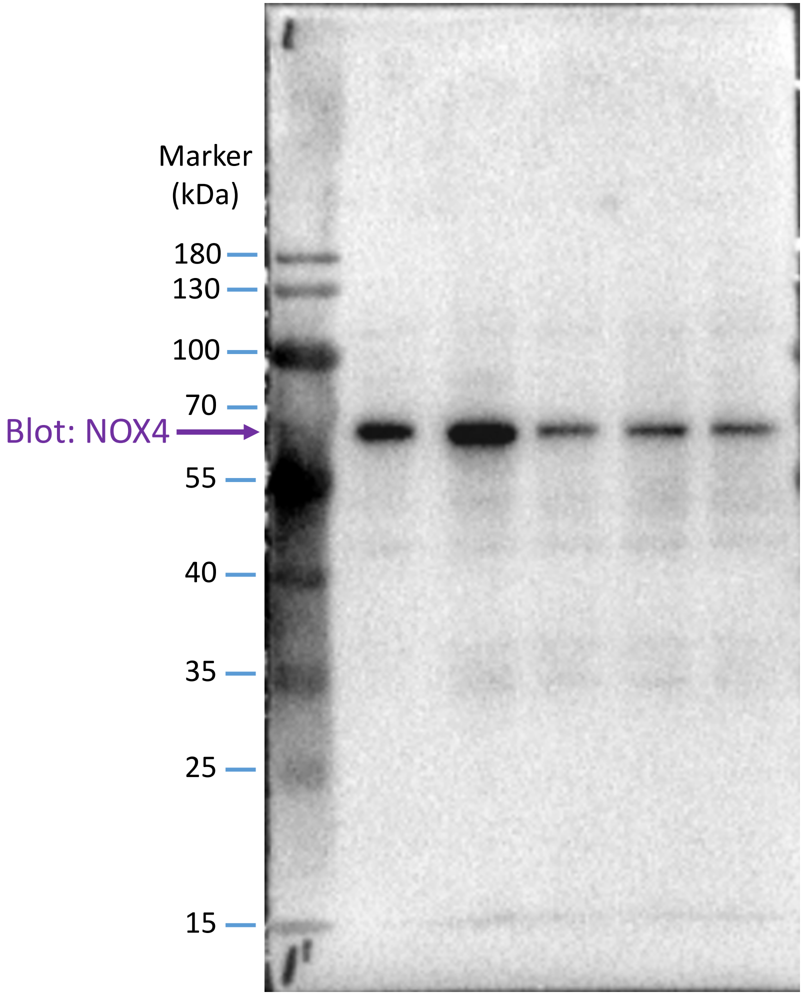

# GAPDH for Figure 4K

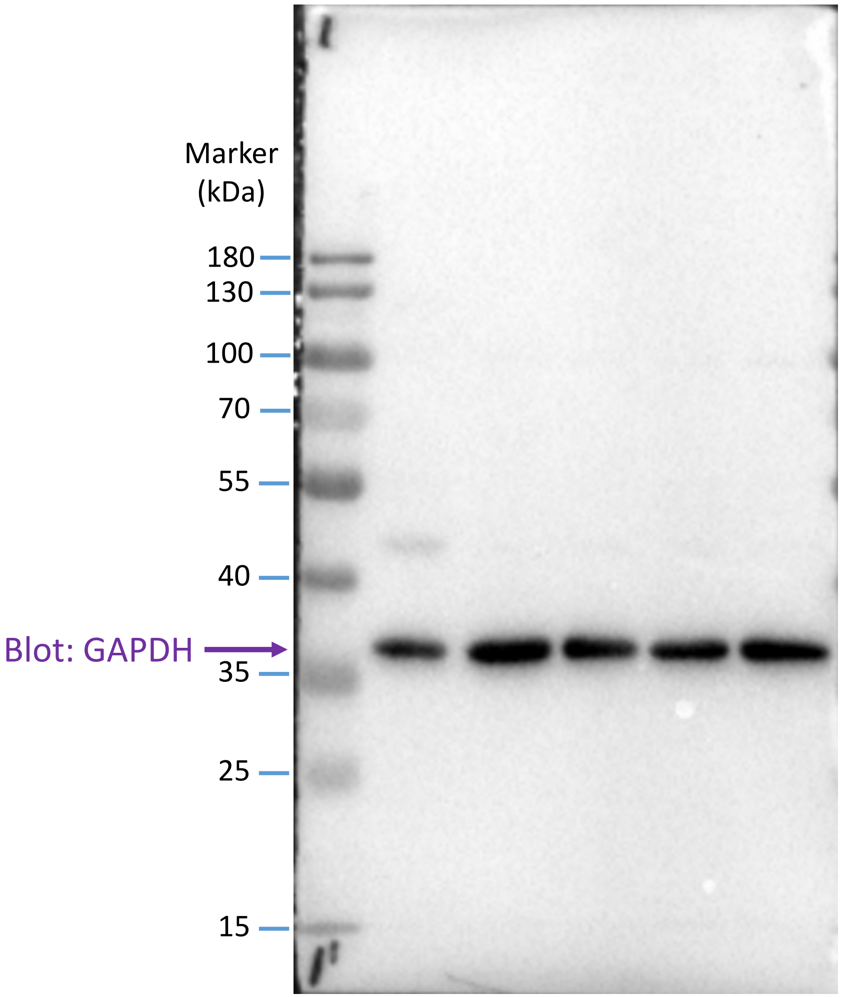

NOX4 for Figure 6K

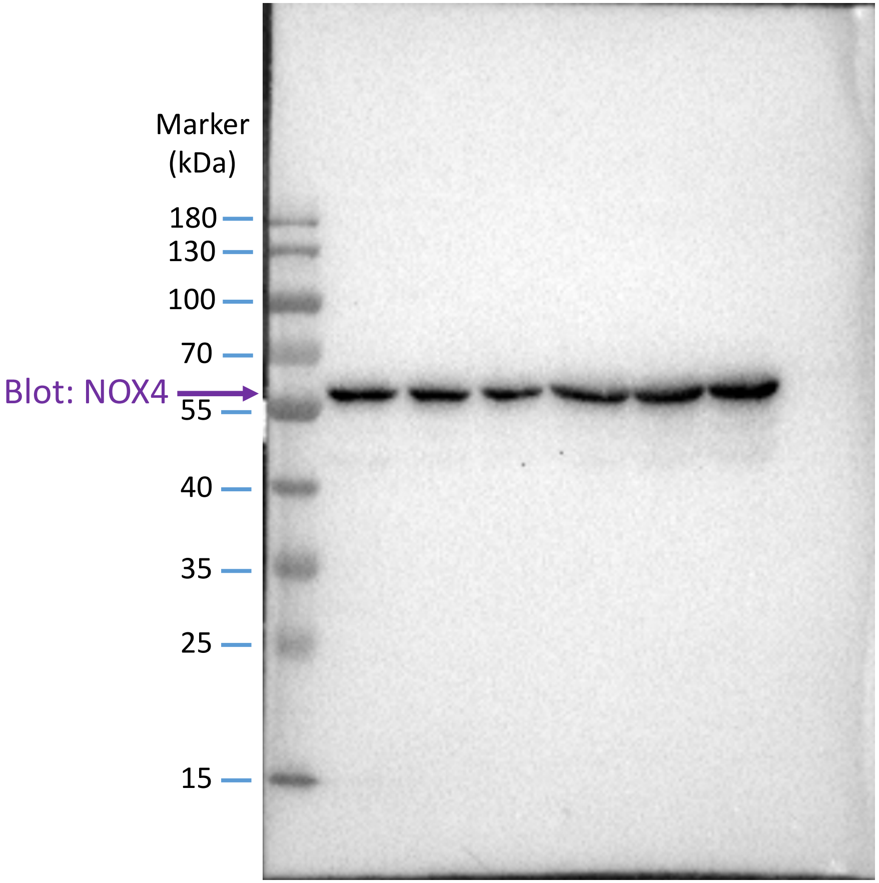

β-actin for Figure 6K

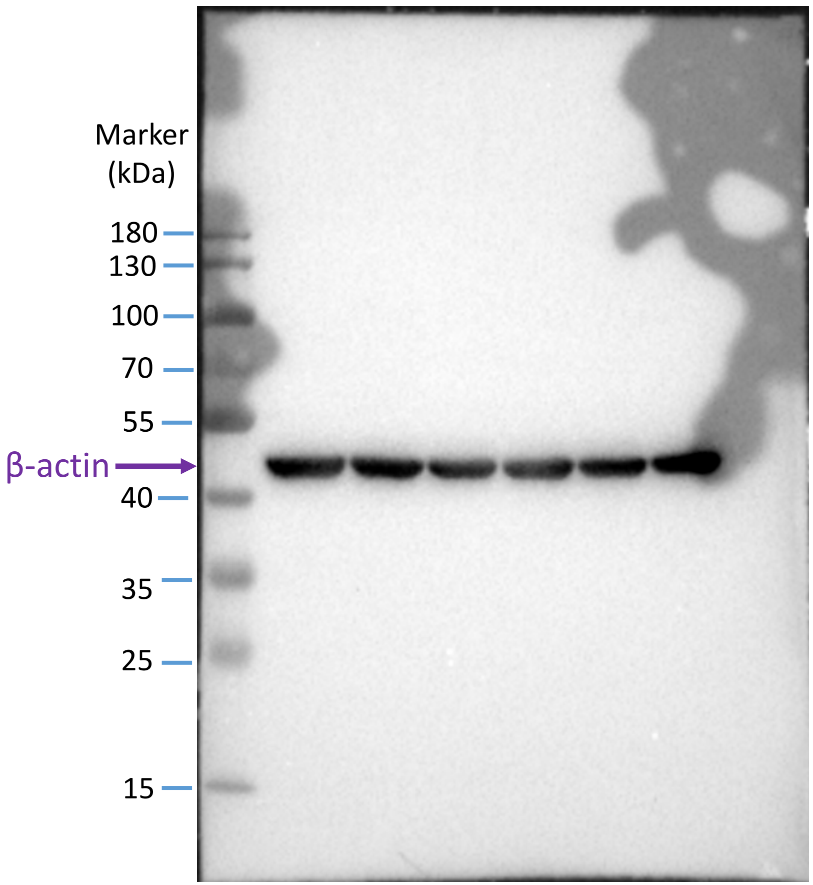

Supplement: Multimedia component 2 [file mmc2.pdf]
